# Preparation of Fe/Ni-MOFs for the Adsorption of Ciprofloxacin from Wastewater

**DOI:** 10.3390/molecules28114411

**Published:** 2023-05-29

**Authors:** Fuhua Wei, Kui Wang, Wenxiu Li, Qinhui Ren, Lan Qin, Mengjie Yu, Zhao Liang, Meng Nie, Siyuan Wang

**Affiliations:** 1 College of Chemistry and Chemical Engineering, Anshun University, Anshun 561000, China; 2 Institute of Micro/Nano Materials and Devices, Ningbo University of Technology, Ningbo 315211, China; 3 State Key Laboratory of Advanced Design and Manufacturing for Vehicle Body, College of Mechanical and Vehicle Engineering, Hunan University, Changsha 410082, China

**Keywords:** metal–organic frameworks, antibiotics, adsorption

## Abstract

This work studies the use of Fe/Ni-MOFs for the removal of ciprofloxacin (CIP) in wastewater. Fe/Ni-MOFs are prepared by the solvothermal method and characterized by X-ray diffraction (XRD), a scanning electron microscope (SEM), Fourier transform infrared spectroscopy (FT-IR), and a thermal gravimetric analyzer (TG). Under the conditions of the concentration of 50 ppm, a mass of 30 mg, and a temperature of 30 °C, the maximum adsorption capacity of ciprofloxacin removal within 5 h was 232.1 mg/g. The maximum removal rate was 94.8% when 40 mg of the Fe/Ni-MOFs was added to the solution of 10 ppm ciprofloxacin. According to the pseudo-second-order (PSO) kinetic model, the R^2^ values were all greater than 0.99, which proved that the adsorption theory of ciprofloxacin by Fe/Ni-MOFs was consistent with the practice. The adsorption results were mainly affected by solution pH and static electricity, as well as other factors. The Freundlich isotherm model characterized the adsorption of ciprofloxacin by Fe/Ni-MOFs as multilayer adsorption. The above results indicated that Fe/Ni-MOFs were effective in the practical application of ciprofloxacin removal.

## 1. Introduction

Water pollution is one of the world’s major environmental problems. China’s rapid economic development, industrial and agricultural development, urban expansion, and other activities have complicated the types and sources of water pollutants, which not only cause great harm to the basin’s water environment but also increase the difficulty in water pollution prevention. The types of water pollution include organic pollution, inorganic pollution, toxic pollution, eutrophication pollution, oil pollution, heat pollution, and pollution by pathogenic microorganisms, etc. [1,2]. The problem of water pollution is becoming increasingly serious due to the rapid development of industry, medicine, and aquaculture and the rising use of antibiotics (such as ciprofloxacin). The abuse and unreasonable discharge of antibiotics are seriously threatening the living environment of human beings, and numerous kinds of antibiotics have been detected in various water resource environments [3,4].

Antibiotics have a strong bactericidal effect [5] and are widely used as antimicrobials to treat and prevent diseases in humans and animals. It is antimicrobial against Haemophilus influenzae, Enterobacter, Streptococcus, Staphylococcus aureus, and Legionella. Due to people’s over-reliance on antibiotics and their widespread use, large amounts of antibiotics enter the environment and become new pollutants, threatening the environment and human health. Antibiotics have been reported to have a very short residence time after entering the body, with only a small proportion being absorbed into the organism for metabolism. Between 60% and 90% of antibiotics are excreted in feces and urine as prototypes or their metabolites [6] and end up in the environment through hospital wastewater, aquaculture wastewater, domestic sewage, and other means, with sewage treatment plants being one of the major sources of environmental antibiotics. It has been reported that more than 85% of CIP frequently enters the environment as raw CIP and its metabolites through sewage treatment, animal manure, etc. In recent years, CIP has been widely detected in environmental media such as water, soil, and plants [7]. The CIP concentration detected in effluent from sewage treatment plants in Brazil was 2378 ng·L^−1^ [8], higher than reported in Wisconsin, Sweden, and in effluents from Chinese sewage treatment plants. The highest CIP concentration detected in effluents from sewage treatment plants in Finland was 4230 ng·L^−1^ [9].The Chinese CIP pollution is also severe. According to the investigation of Changsha, the concentration of CIP is 0.03–0.15 μg·L^−1^ in Xiangjiang River, 0.02~0.34 μg·L^−1^ in Laodao River, and 0.01~0.8 mg·L^−1^ in the sewage treatment plant.

Ciprofloxacin is a quinolone antibiotic with broad-spectrum antibacterial activity. Its mechanism of action is to inhibit DNA cyclase and play a bactericidal role by destroying the structure of bacterial DNA, preventing cell division [10]. Ciprofloxacin has low toxicity, few side effects, and is widely used in human and veterinary medicine. However, the extensive use of ciprofloxacin is leading to environmental pollution, including soil and water contamination, which can seriously endanger human life and health [11]. Ciprofloxacin is present in wastewater at varying levels, and its presence is already threatening ecosystems and human health. Therefore, the efficient, environmentally friendly, and rapid removal of ciprofloxacin antibiotic pollution in wastewater has become a key issue to be solved [12].

Scientists have explored a variety of methods to remove antibiotics. Treatment for antibiotics in wastewater includes biological, chloride, electrochemical, adsorption, thin film, and microbial degradation methods [13], all of which can remove antibiotics. However, many of these techniques have various problems, such as technical difficulty, high cost, certain risks, low degradation, and selection effects.

Metal–organic frameworks (MOFs) were first reported in the 1990s as a new functional material with an adjustable structure and the advantages of good selectivity, high specific surface areas, and porosities [14]. MOF materials are unlike traditional porous inorganic materials and are widely used in many scientific research fields, such as adsorption and separation [15,16,17], catalysis [18], chemical sensing [19], carbon dioxide capture [20,21], energy storage [22,23], antibiotics [24,25], drug delivery [26,27], the adsorption of heavy metal ions [28,29], organic dyes [30,31,32,33,34,35,36], etc. MOFs also have good application prospects for wastewater treatment. The Yaghi research group completed a large amount of fundamental research into MOF materials [37], particularly MOF-5, which is considered a milestone in metal-organic skeleton materials [38]. With the continuous exploration and application of MOF materials, an increasing number of studies have been conducted on their use in the field of environmental pollution [39,40,41]. MOF materials can not only detect pollutants in water but also adsorb them, and their significant application value is gradually emerging [42].

While monomeric MOF materials have poor pollutant removal effects, the synergistic phenomenon between metals in polymetallic MOF materials results in a better pollutant removal effect [39]. In this paper, metal synergism is employed to prepare Fe/Ni-MOFs with iron and nickel ions as metal ion sources and 1,3,5-phthalic acid as the organic ligand under solvothermal action. The use of this material for the removal of ciprofloxacin is then studied for use in wastewater treatment.

## 2. Results

### 2.1. Material Characterization

**Figure 1:**
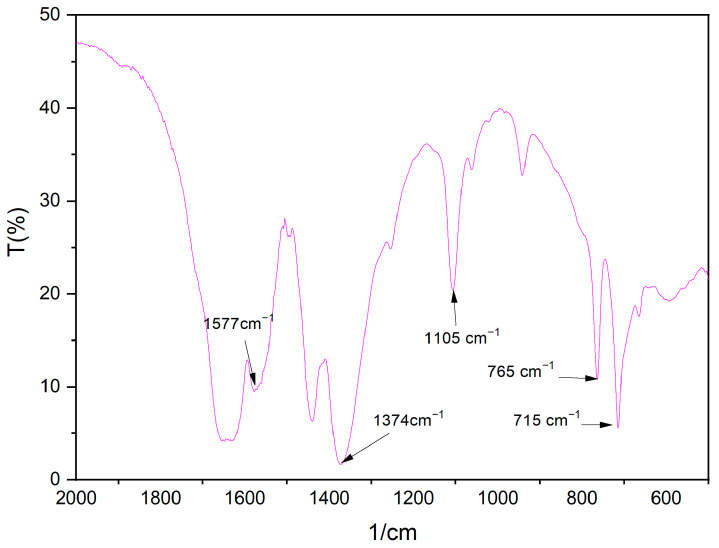
FTIR spectrum of Fe/Ni-MOFs.

The FTIR shown in Figure 1 indicates there are strong absorption peaks at 1577 and 1374 cm^−1^, respectively. This is largely attributed to the delocalization of the carboxyl group of the organic chain during the reaction, rendering the two C-O bonds in an equal state. Strong absorption peaks appear between 1620–1550 and 1420–1300 cm^−1^, which indicate that carboxylic acid can react with metal salt [43]. In total, 715 cm^−1^ is the substitution of 1,3,5 on the organic ligand, 765 cm^−1^ is the benzene ring C-H plane bending vibration, and 1105 cm^−1^ is the stretching vibration of C-O bond.

**Figure 2:**
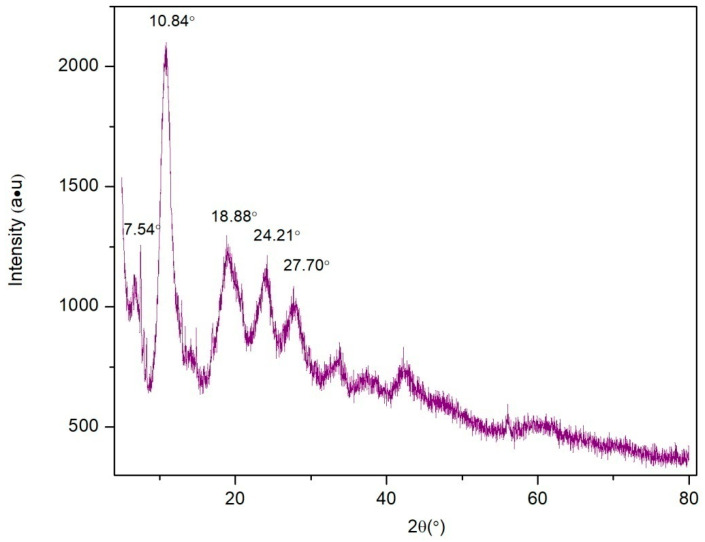
XRD diffractogram of Fe/Ni-MOFs.

**Figure 3:**
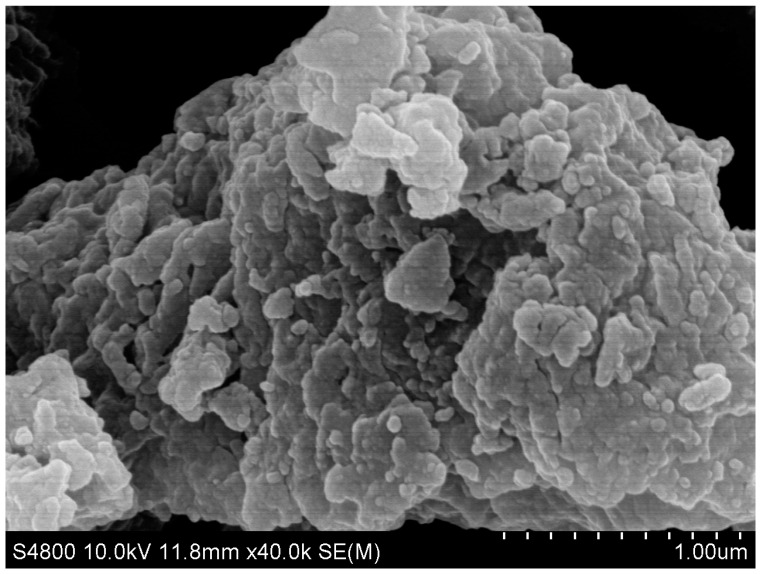

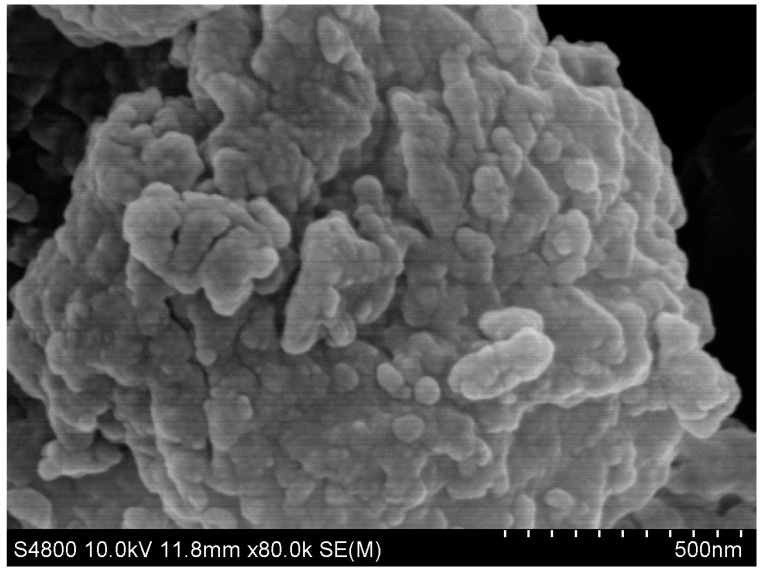
SEM micrographs of Fe/Ni-MOFs.

As shown in Figure 2, the XRD characteristic peaks of the Fe/Ni-MOFs mainly appear at 10.8°, 18.9°, 24.2°, and 27.7°, and the diffraction peaks of the Fe/Ni-MOFs are sharp and strong. According to the results, the Brunauer–Emmett–Teller surface area is 497.9 m^2^/g, the average adsorption pore diameter is 2.03 nm, and the average particle size is 12.0501 nm, illustrating the mesoporous nature of the material. Combined with the SEM image (as shown in Figure 3) and BET testing outcome, it can be proved that the Fe/Ni-MOFs have high crystallinity and good dispersion. This is mainly because the interaction between organic ligands and metal ions is weakened or even disappears while the deprotonation of organic ligands is enhanced, which promotes crystal growth in organic solvents.

**Figure 4:**
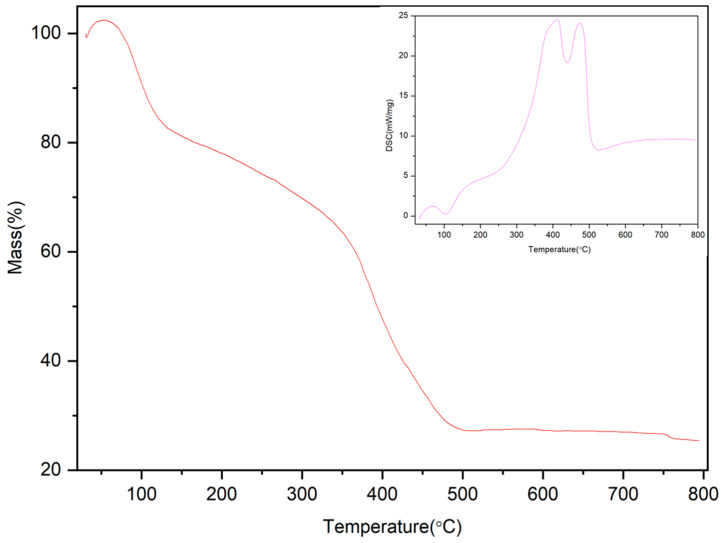
TG curve of Fe/Ni-MOFs.

The stability of the material is one of the factors affecting its performance. It can be seen from Figure 4 that the mass increases by 2.4% at the beginning, which is mainly because the material is porous and can absorb part of the gas. As illustrated in Figure 4, there are two main stages of material loss. The first stage occurs between 20 and 132 °C, which is largely attributed to the solvent contained in the material, and the loss is 17.4% (1.015 mg). In the second stage, the organic chains in the material are broken, and the structure begins to collapse until 506 °C, at which stage 35.7% (2.083 mg) is lost; at 800 °C, there is 25% (1.459 mg)residue [44,45].

### 2.2. Removal of Ciprofloxacin by Fe/Ni-MOFs

**Figure 5:**
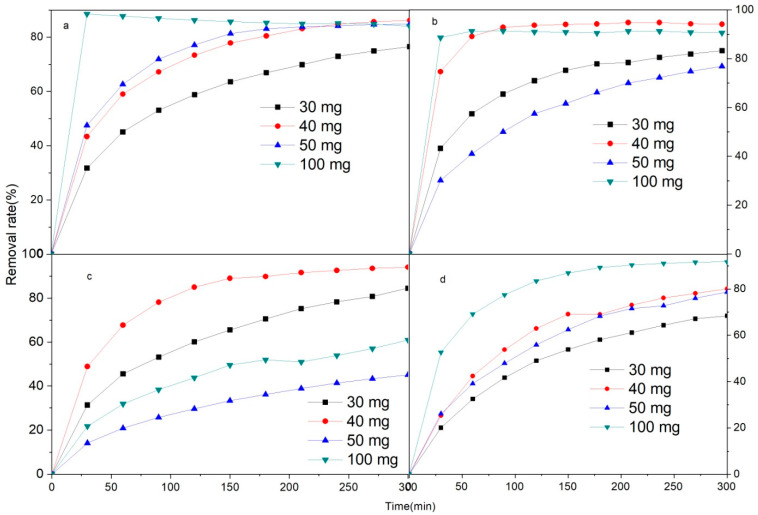
Removal rate of Fe/Ni-MOFs ((**a**) 5 ppm; (**b**) 10 ppm; (**c**) 20 ppm;(**d**) 30 ppm).

Different amounts of the Fe/Ni-MOFs were tested to verify their ability to remove antibiotics (ciprofloxacin). Figure 5 shows that the removal first increases and then decreases with increasing mass at a constant concentration. Up to 94.13% removal was achieved with a 10 mg/L concentration of ciprofloxacin and a 40 mg mass of the Fe/Ni-MOFs. For ciprofloxacin concentrations of 20 mg/L and 50 g mass Fe/Ni-MOFs, removal was the worst, with a removal rate of only 45.07%. A contributing factor to this may have been the large number of the Fe/Ni-MOFs covering the active sites, reducing the adsorption capacity.

The removal effect of the Fe/Ni-MOFs on ciprofloxacin was further studied by simulating the first and second kinetics. The results are shown in Figure 5, and the kinetic formula is: [46,47,48]

(1) $$\ln\frac{C_{t}}{C_{0}}=k_{1}t$$

(2) $$\frac{t}{q_{t}}=\frac{t}{q_{e}}+\frac{1}{k_{2}q_{e}^{2}}$$

where k is the kinetic reaction constant (min), q_t_ is the adsorption amount of MOFs per unit time, and q_e_ (mg/g)is the adsorption amount of MOFs at equilibrium.

**Figure 6:**
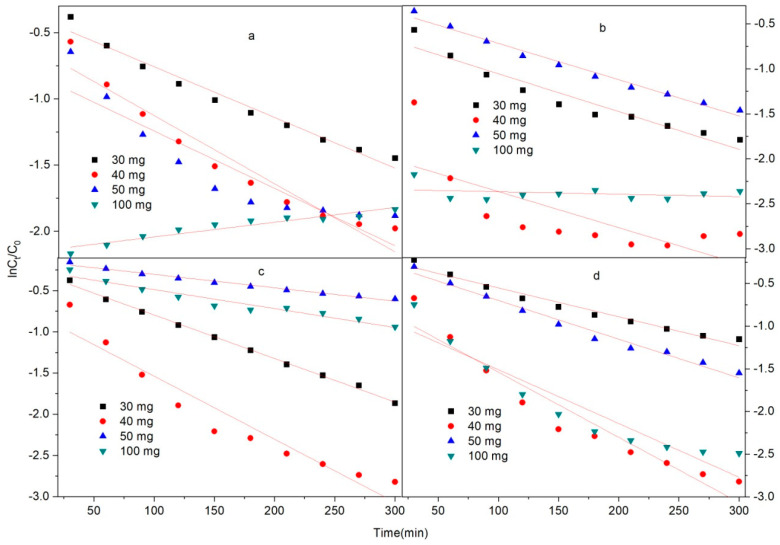
The pseudo-first-order kinetic model for the adsorption of ciprofloxacin over the Fe/Ni-MOFs: (**a**) 5 ppm; (**b**) 10 ppm; (**c**) 20 ppm; (**d**) 30 ppm.

**Figure 7:**
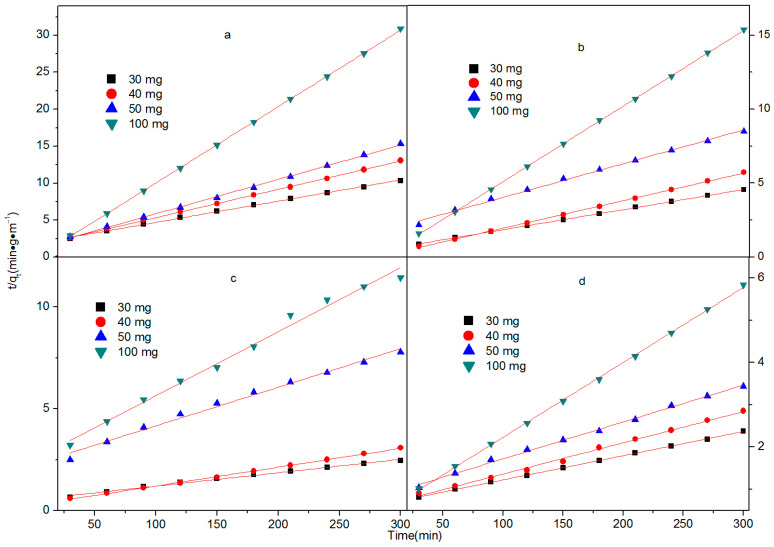
The pseudo-second-order kinetic model for the adsorption of ciprofloxacin over the Fe/Ni-MOFs: (**a**) 5 ppm; (**b**) 10 ppm; (**c**) 20 ppm; (**d**) 30 ppm.

As illustrated in Figure 6 and Figure 7 and Table 1, the R^2^ of the second kinetics is basically around 0.99, which is better than that of the first kinetics. This result indicates that the removal of ciprofloxacin by Fe/Ni-MOFs is mainly by chemisorption, and the removal ability of ciprofloxacin by Fe/Ni-MOFs is essentially consistent with the effect in practice.

To explore the effect of temperature on the removal of ciprofloxacin by Fe/Ni-MOFs, the reaction temperatures were controlled at 30, 40, and 50 °C. The data were further analyzed by the Langmuir and Freundlich isotherm models. At the same time, in order to ensure the theory was close to practice, the removal efficiency was calculated by the following formulas:

(3) $$\frac{C_{e}}{q_{e}}=\frac{C_{e}}{q_{\max}}+\frac{1}{K_{L}q_{\max}}$$

(4) $${\mathrm{lnq}}_{e}=\frac{1}{n}{\mathrm{lnC}}_{e}+{\mathrm{lnK}}_{f}$$

**Figure 8:**
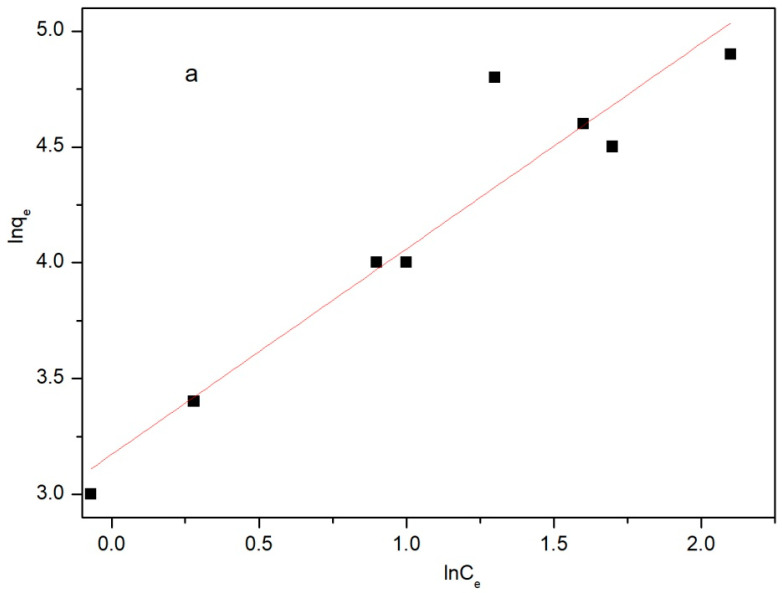

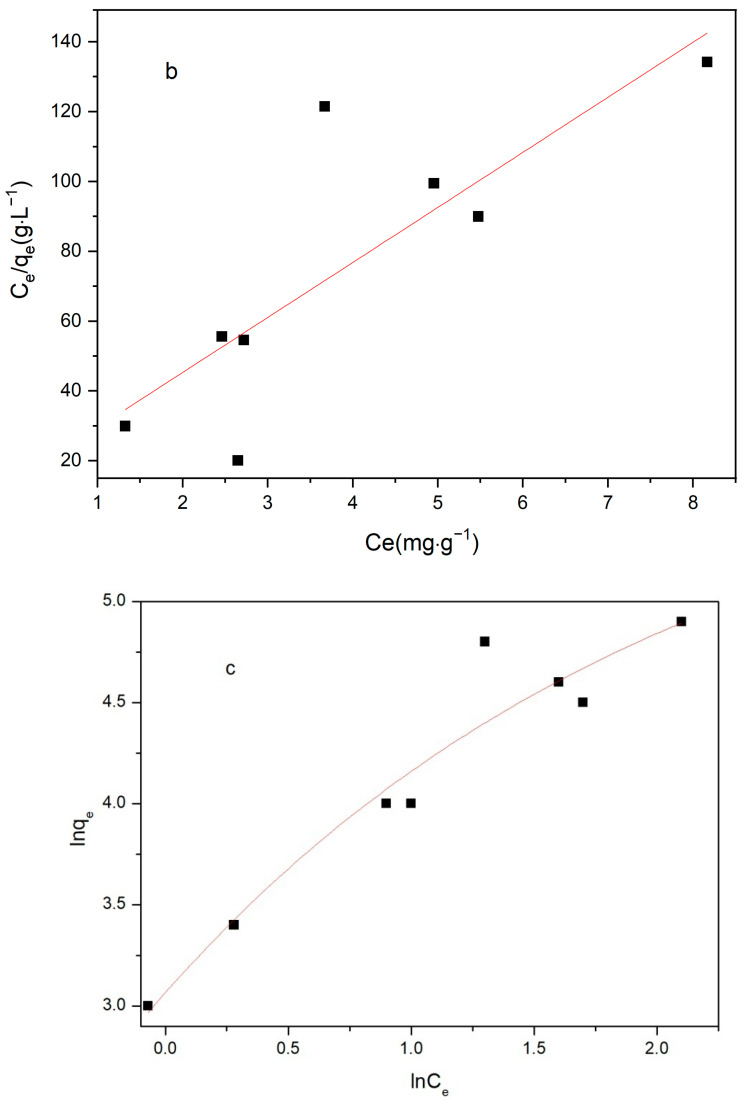

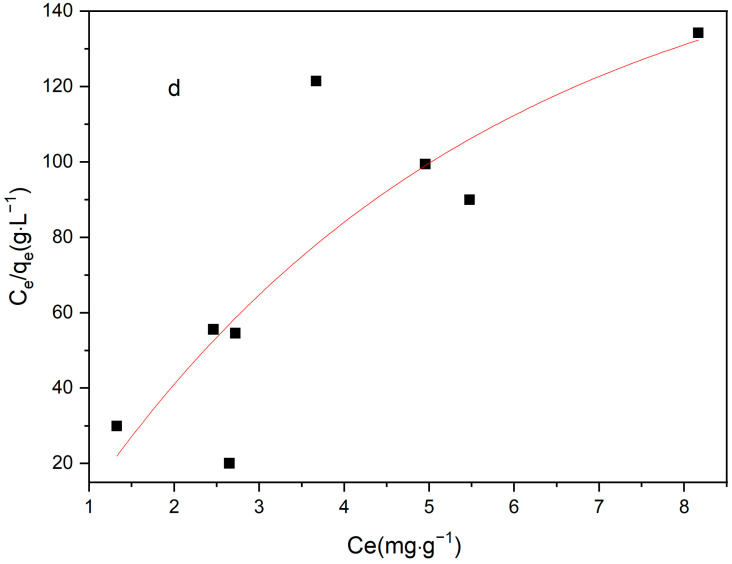
Adsorption isotherm of ciprofloxacin onto Fe/Ni-MOFs at room temperature: (**a**) Freundlich; (**b**) Langmuir; (**c**) Freundlich (non-linear); (**d**) Langmuir(non-linear).

The effect of temperature on adsorption was further analyzed by adding 30 mg of the Fe/Ni-MOFs and 30 mg/L ciprofloxacin solution at the same conditions. The results are provided in Figure 8 and Table 2. As illustrated, Freundlich isotherm is more suitable to describe the adsorption of the Fe/Ni-MOFs to ciprofloxacin. The Fe/Ni-MOFs’ adsorption of ciprofloxacin is a typical, mainly physical adsorption.

**Figure 9:**
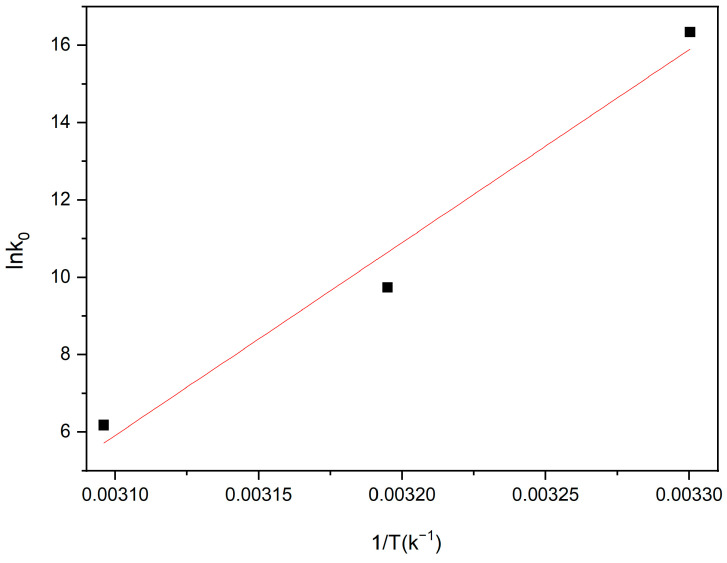
Van ‘t Hoff plots showing the ΔH and ΔS of ciprofloxacin adsorption over Fe/Ni-MOFs.

The adsorption mechanism of ciprofloxacin by Fe/Ni-MOFs was then analyzed by evaluating the thermodynamic equilibrium constant, Gibbs free energy, and the Van ‘t Hoff equation, according to the following formula:

(5) $${\mathrm{lnK}}_{0}=\frac{\Delta S^{0}}{R}-\frac{\Delta H^{0}}{\mathrm{RT}}$$

(6) $$\Delta G^{0}=-{\mathrm{RTlnK}}_{0}$$

(7) $$K_{0}=\frac{q_{e}}{c_{e}}$$

where R is the gas constant (8.314 Jmol^−1^ k^−1^), and K_0_ is the Langmuir adsorption constant (L mol^−1^). To obtain the values of ΔH^0^ and ΔS^0^, a linear plot of lnK_0_ versus 1/T is constructed, as shown in Figure 9. The ΔH^0^ and ΔS^0^ are then calculated from the Van ’t Hoff plot, as shown in Table 3.

As illustrated in Figure 9 and Table 3, ΔG^0^ and ΔH^0^ are both negative, indicating that the adsorption of ciprofloxacin by Fe/Ni-MOFs is spontaneous and exothermic. The enthalpy of chemisorption ranges from 84 to 420 kJ mol^−1^, and physical adsorption occurs when the enthalpy changes by less than 84 kJ mol^−1^. Therefore, the Fe/Ni-MOFs’ adsorption of ciprofloxacin is a typical physical adsorption, and entropy and enthalpy changes are the main influencing factors of the adsorption [49,50,51].

In summary, the enhanced effect of the Fe/Ni-MOFs on ciprofloxacin removal is mainly due to the factors discussed below. As Fe/Ni-MOFs are porous materials, ciprofloxacin can be adsorbed by the pores of the Fe/Ni-MOFs. Fe/Ni-MOFs may have some unreacted groups, and the Zeta potential characterization indicates that the charge in the Fe/Ni-MOFs is −9.98 mv in the water, which can be adsorbed electrostatically. As Fe/Ni-MOFs and ciprofloxacin both have benzene rings, the two molecules may be bound together by π-π adsorption. Fe/Ni-MOF materials and ciprofloxacin also have the capacity to form hydrogen bonds [36,37,38,39,40]. In summary, the adsorption of ciprofloxacin by Fe/Ni-MOF materials is a combination of chemisorption and physical adsorption, which enhances the removal effect.

**Figure 10:**
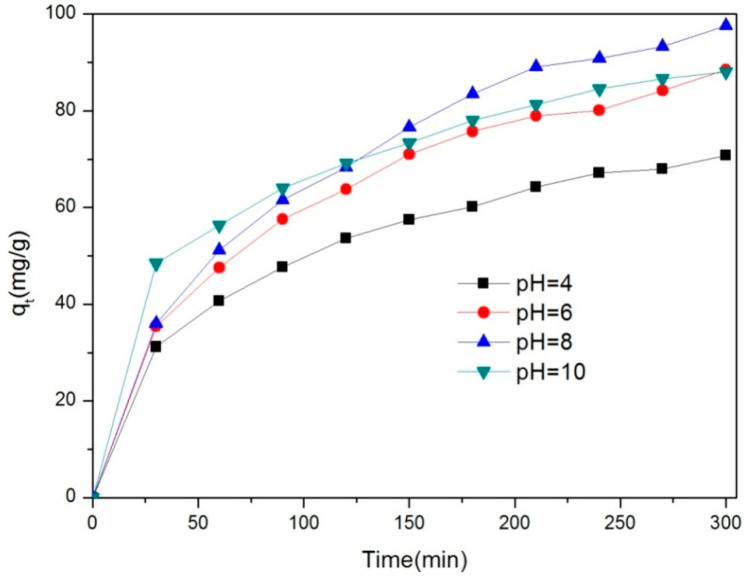
Effect of pH on the adsorption amount of ciprofloxacin.

The effect of pH on ciprofloxacin was studied by adding 30 mg of the Fe/Ni-MOFs to 30 mg/L of ciprofloxacin at different pH levels, whereby the structures of the Fe/Ni-MOFs are destroyed in strong acid or alkali solutions. As shown in Figure 10, with increasing pH, the adsorption capacity first increases and then decreases. The adsorption is the best.

The adsorption effects of the Fe/Ni-MOFs for the removal of ciprofloxacin were then compared with other adsorbents. As shown in Table 4, Fe/Ni-MOFs are effective for ciprofloxacin removal.

## 3. Discussion

Due to people’s over-reliance on antibiotics and their widespread use, large amounts of antibiotics enter the environment and become new pollutants, threatening the environment and human health. As the antibiotic contamination of aqueous solutions is becoming more severe, and ciprofloxacin contamination, in particular, is increasing every year, we focus on the removal of ciprofloxacin from aqueous solutions in this paper. There are many methods to remove organic contaminants, and more theoretical studies include photocatalysis and adsorption, but the results are not good. In this paper, we use Fe/Ni-MOFs to remove ciprofloxacin with good results and investigate its theory using kinetic models and adsorption isotherms.

### 3.1. Synthesis of Fe/Ni-MOFs

Metal-organic framework materials are usually single metal ion sources, and the treatment effect is relatively poor, whereas bimetal ion sources have good synergy, resulting in better effects (see Figure 5 and Table 4). In the process of the preparation of Fe/Ni-MOFs, the unreacted organic chains are removed by washing with an organic solvent, whereas the unreacted metal ions are removed by washing with water.

The dried Fe/Ni-MOFs were characterized by their structures (as shown in Figure 1, Figure 2, Figure 3 and Figure 4). Infrared could determine whether functional groups are involved in the reaction, and infrared spectroscopy (as shown in Figure 1) showed that the carboxyl group of the organic chain formed two symmetric C-O bonds after declaration, thus proving the formation of new substances. XRD and SEM images could demonstrate that the material had different morphologies and structures. The TG diagram was mainly used to test the thermal stabilities of the Fe/Ni-MOFs. As can be seen in Figure 4, the masses of the Fe/Ni-MOFs increased slightly in the first phase, mainly due to the porous structures of the Fe/Ni-MOFs. When N_2_ was introduced, the Fe/Ni-MOFs slightly increased in mass. The masses of the Fe/Ni-MOFs increased slightly as the gas entered the pores. When the temperature reached 350 °C, the frame structure began to be destroyed, which proved that the Fe/Ni-MOFs were stable below 350 °C, and the skeleton structure was basically destroyed when the temperature reached 506 °C.

### 3.2. CIP Removal by Fe/Ni-MOFs

At room temperature, Fe/Ni-MOFs of various masses were added to the CIP solution at various concentrations, stirred on a magnetic agitator, and sampled every 30 min. The absorbance variations in the samples were analyzed in an ultraviolet analyzer, and the specific data were obtained following the standard curve, which was used in the removal rate. The effects of the concentration of the CIP solution, the masses of the Fe/Ni-MOFs, the pH value of the CIP solution, and the temperature were discussed, and the kinetic model, the isothermal adsorption equation, and the Van ‘t Hoff equation were used to study the CIP adsorption.

As can be seen in Figure 5, the removal rate increases with the mass of the Fe/Ni-MOFs. As the adsorption of the Fe/Ni-MOFs onto CIP is primarily attributed to physical adsorption, the addition of 100 mg of the Fe/Ni-MOFs to a 5 ppm CIP solution leads to rapid attainment of equilibrium, followed by slow desorption, resulting in reduced removal efficiency (as depicted in Figure 5a). When 100 mg of the Fe/Ni-MOFs is added to 10 ppm CIP solution, the maximum removal rate can reach 94.1% (as shown in Figure 5b). When 100 mg of the Fe/Ni-MOFs is added to 20 ppm CIP solution, after 5 h, the removal rate is only 60.8% (as shown in Figure 5c), which is mainly due to the reduced removal rate due to excessive Fe/Ni-MOFs coverage of the active site.

As can be seen from the kinetic model, the correlation coefficients of the second kinetic model are 0.98909 and 0.98959 for masses as small as 20 ppm and 50 and 100 mg, and all other correlation coefficients are larger than 0.99. From this, it can be seen that the removal of the CIP by the Fe/Ni-MOFs is appropriate for the second kinetic model. The first kinetic model’s results show that the correlation coefficient is good only for concentrations of 20 ppm and masses of 30 mg. Therefore, the removal of CIP by Fe/Ni-MOFs is mainly based on chemical adsorption.

As can be seen from the adsorption isotherms of the Fe/Ni-MOFs on the CIP, both the Freundlich and Langmuir isotherms have correlation coefficients below 0.99, and the Freundlich isotherm has a correlation coefficient 0.98. Relatively above the Langmuir isotherm of 0.89, it can be seen that the CIP adsorption of the Fe/Ni-MOFs is dominated by physical adsorption. According to the Van ‘t Hoff equation, the obtained ΔG^0^ and ΔH^0^ values are negative when ΔH^0^ ranges from 84~420 kJ/mol for chemical adsorption and when ΔH^0^ < 84 kJ/mol, which is typical for physical adsorption, according to which the CIP adsorption of the Fe/Ni-MOFs is physical. It is shown that the adsorption capacities of the Fe/Ni-MOFs on the CIP decrease with increasing temperature. It is concluded that the thermal energy of the CIP molecules is lower at lower temperatures, which reduces the probability of collisions with the Fe/Ni-MOFs. As the temperature increases, the thermal motion of the CIP molecules is facilitated, and the probability of the adsorption of the CIP molecules by Fe/Ni-MOFs increases. However, when the temperature is too high, the thermal motion of the molecules is too strong, and the desorption rate of Fe/Ni-MOFs onto the CIP molecules is larger than the adsorption rate, which eventually leads to a decrease in the adsorption capacity with increasing temperature. Moreover, the pore structures of the Fe/Ni-MOFs are strongly temperature dependent. As the temperature increases, the pore structures increase due to the effects of thermal expansion and cold contraction. At this point, the adsorption of the CIP molecules by the Fe/Ni-MOFs increases with temperature. Eventually, the adsorption capacity decreases.

The pH of the 30 ppm CIP solution was adjusted to 4, 6, 8, and 10 before adding 30 mg of the Fe/Ni-MOFs with stirring on a magnetic agitator. Samples were taken every 30 min to detect absorbance. At strong acid and substrate conditions, the adsorption capacity was low. To compare the CIP adsorption capacities of the Fe/Ni-MOFs with those of other materials of the same type, the Fe/Ni-MOFs had the highest adsorption capacities on the CIP, which demonstrated that the Fe/Ni-MOFs had the best CIP removals.

In summary, as Fe/Ni-MOFs are porous materials, and the presence of benzene rings between the Fe/Ni-MOFs and CIP leads to the better adsorption of Fe/Ni-MOFs.

## 4. Research Methods

### 4.1. Experimental Raw Material

The raw materials of seven ferrous sulfate hydrate (99.0%), nickel acetate (II) four hydrate (99.9%), and 1,3,5-benzyl formate three were obtained from Shanghai Aladdin Biochemical Technology Co., Ltd. (Shanghai, China). Additionally, antibiotic ciprofloxacin (98%) was obtained from Shanghai MacLean Biochemical Technology Co., Ltd. (Shanghai, China).

### 4.2. Preparation of Fe/Ni-MOFs

The preparation of Fe/Ni-MOFs by solvothermal method has been reported. Ferrous sulfate heptahydrate (556.02 mg, 2 mmol), nickel acetate (497.68 mg, 2 mmol), and 1,3,5-phthalic acid (420.28 mg, 2 mmol) were dissolved in N,N-dimethylformamide, respectively. When completely dissolved, they were combined and stirred with a magnetic stirrer for 30 min to mix thoroughly. The mixture was then transferred to a reaction kettle and put into a constant temperature drying oven for 12 h at 150 °C. At the end of the reaction, the individual reactants were removed, cooled to room temperature, filtered, washed with a small amount of DMF three times, washed with ethanol three times, and, finally, washed with water three times. The solid was transferred to the oven at 80 °C for 12 h to dry, i.e., the raw materials.

### 4.3. Characterization of Fe/Ni-MOFs

The fabricated Fe/Ni-MOF materials were used for structural characterization. The main characterization methods were as follows: structural and morphology characterization were performed by X-ray diffraction (XRD, D-5000, Siemens, Munich, Germany, Cu Ka) and a field emission scanning electron microscope (FESEM, JSM-6700F, Gansu Jingpu Testing Technology Co., Ltd., Lanzhou, China). The thermogravimetry (TG) curve of the particles was recorded by a NETZSCH STA 449C thermal analyzer (Shenzhen Taili Instrument Co., Ltd., Shenzhen, China) in a nitrogen (N_2_) atmosphere, heating from 0 °C to 800 °C at a heating rate of 5 °C min^−1^. After mixing the Fe/Ni-MOFs with KBr, absorption spectra were tested in the range 500–3000 cm^−1^ on a Shimadzu by Fourier transform infrared spectroscopy (FT-IR, IR Tracer-100, Shimadzu Enterprise Management (China) Co., Ltd., Shanghai, China).The specific surface areas, pore volumes, pore sizes, and pore distributions of the Fe/Ni-MOFs were tested by an automated specific surface area and a micropore voidage and chemisorption analyzer (ASAP2020M + C, Mike Instruments, Shanghai, China). Fe/Ni-MOFs were dispersed in an aqueous solution to determine their Zeta potentials (mV).

### 4.4. Removal of Ciprofloxacin

The abilities of the Fe/Ni-MOFs to eliminate the antibiotic ciprofloxacin were tested at room temperature in a 250 mL beaker. In the experiment, 30, 40, 50, and 100 mg of the Fe/Ni-MOFs were added to ciprofloxacin solutions at 5, 10, 20, and 30 mg/L concentrations, respectively. Under the action of natural light, the mixtures were placed in a magnetic stirrer for stirring, and samples were taken every 30 min. Finally, the absorbance data of the samples were measured by a UV-Vis spectrophotometer, and λmax = 277 nm [55]. The concentration was calculated by the absorbance, and the removal rate and adsorption capacity of the ciprofloxacin at different time intervals were obtained.

(8) $$C\left(\%\right)=\frac{C_{0}-C_{t}}{C_{0}}\times 100\%$$

(9) $$q_{e}=\frac{\left(C_{0}-C_{e}\right)V}{m}$$

where C_0_ (mg/L) represents the initial concentration, Ct (mg/L) represents the concentration at equilibrium, V(mL) represents the volume of solution, and m(mg) represents the mass of MOFs.

### 4.5. Effect of pH on Adsorption of Ciprofloxacin by Fe/Ni-MOFs

The pH was adjusted with NaOH (0.1 mol/L) and hydrochloric acid (0.1 mol/L) in 200 mL of 30 ppm ciprofloxacin solution. In total, 30 mg of the Fe/Ni-MOFs was added to the adjusted solution, stirred, and sampled every 30 min. The concentration of ciprofloxacin solution was analyzed by a UV spectrometer (Shanghai Haoliang Photoelectric Equipment Co., Ltd., Shanghai, China).

## 5. Conclusions

This work successfully prepared Fe/Ni-MOF materials using the solvothermal method. The structures of the prepared materials were then characterized by SEM, FT-IR, TG, etc., and the characterized materials were analyzed for the removal of ciprofloxacin by the Fe/Ni-MOFs. According to the results, under the conditions of a concentration of 50 ppm, a mass of 30 mg, and a temperature of 30 °C, the maximum adsorption capacity of the ciprofloxacin removal within 5 h was 232.1 mg/g. The kinetic model and adsorption isothermal showed that the Fe/Ni-MOFs conformed to the chemisorption of ciprofloxacin. The findings demonstrated that the theoretical and practical effects of the Fe/Ni-MOFs on ciprofloxacin were consistent. Therefore, the Fe/Ni-MOFs had good development prospects for ciprofloxacin removal in practical applications.

## Figures and Tables

**Table 1:** Kinetic parameters for the adsorption of ciprofloxacin over the Fe/Ni-MOFs.

Con (ppm)	Mass (mg)	Pseudo-Second-Order Kinetics		Pseudo-First-Order Kinetics	
		K(g·(mg·min)^−1^)	R^2^	K(L·min^−1^)	R^2^
5	30	0.02874	0.99824	−0.00382	0.97152
	40	0.03831	0.99973	−0.00513	0.93588
	50	0.04643	0.99882	−0.00432	0.81166
	100	0.10336	0.99983	0.00111	0.89982
10	30	0.01357	0.9997	−0.00421	0.92322
	40	0.01861	0.99955	−0.00402	0.50108
	50	0.02282	0.99637	−0.00404	0.98182
	100	0.05111	0.99993	−0.000273	-
20	30	0.00657	0.99348	−0.00528	0.99668
	40	0.00924	0.99914	−0.00765	0.9167
	50	0.01895	0.98909	−0.00162	0.97983
	100	0.03144	0.98959	−0.0023	0.9383
30	30	0.00573	0.99967	−0.00339	0.96968
	40	0.00739	0.99588	−0.00765	0.9167
	50	0.00872	0.99683	−0.00454	0.97988
	100	0.01774	0.99940	−0.0063	0.88405

**Table 2:** Adsorption isotherm parameters of ciprofloxacin onto MOFs at room temperature.

T (K)	Langmuir Isotherm		Freundlich Isotherm		
	k	R^2^	K_f_ (mg/g (L/mg)^1/n^)	n	R^2^
293 (linear)	0.06343	0.62107	40.9355	1.1267	0.89451
293 (non-linear)	-	0.59199	-	-	0.90406

**Table 3:** The thermodynamic parameters of ciprofloxacin adsorption onto Fe/Ni-MOFs.

T (K)	ΔG^0^ (KJ/mol)	ΔH^0^ (−Slope × R) (KJ/mol)	S^0^ (Intercept × R) (J/mol/K)
298	−46.2	−414.6	−1236

**Table 4:** Comparison of the adsorption of ciprofloxacin with other adsorbents.

Adsorbent	q_max_ (mg g^−1^)	References
ZIF-67-NO_3_	86.4	[52]
ZIF-67-Cl	92.3	
ZIF-67-SO_4_	93.5	
ZIF-67-OAc	80.1	
ZIF-8-leaf	86.5	
UIO-66	57.5	
ZIF-8-Cube	69.2	
Fe_3_O_4_/C	98.28	[53]
SiO_2_ Nanoparticles	59.28	[54]
Fe/Ni-MOFs	232.1	This work

## Data Availability

The data presented in this study are available on request from the corresponding author.

## Sample Availability

Samples of the compounds are available from the authors.
